# A robust cross-sectional assessment of the impacts of COVID-19 pandemic on the prevalence of female genital mutilation among 0–14 years old girls in Nigeria

**DOI:** 10.1177/17455057241311948

**Published:** 2025-05-26

**Authors:** Corentin Visée, Camille Morlighem, Chibuzor Christopher Nnanatu

**Affiliations:** 1 Department of Geography, University of Namur, Namur, Belgium; 2 Institute of Life, Earth and Environment (ILEE), University of Namur, Namur, Belgium; 3 Fonds National de la Recherche Scientifique (F.R.S-FNRS), Brussels, Belgium; 4 WorldPop, School of Geography and Environmental Science, University of Southampton, Southampton, UK; 5 Department of Statistics, Nnamdi Azikiwe University, Awka, Nigeria

**Keywords:** Bayesian hierarchical modelling, spatial analysis, DHS, MICS, social norms, FGM abandonment

## Abstract

**Background:**

Female genital mutilation (FGM) is a human rights violation that still affects more than 3 million girls aged 0–14 years each year. To achieve the Sustainable Development Goal 2030 agenda, efforts have been made at the local, national and international levels to end the practice by the year 2030. However, the recent COVID-19 pandemic may have reversed the progress made due to increased rates of early marriage of girls, violence against children and school closures during lockdowns. Although some surveys have examined changes in FGM prevalence over the COVID-19 period, changes at the national and sub-national levels among 0–14 years old girls have not been quantified.

**Objectives:**

This study aimed to understand the potential impacts of the COVID-19 pandemic on the likelihood of FGM among girls aged 0–14 years, and whether it affected progress towards the elimination of FGM.

**Design:**

We used Bayesian hierarchical regression models implemented within the integrated nested Laplace approximations frameworks.

**Methods:**

We modelled the likelihood and prevalence of FGM among girls aged 0–14 years before and after the COVID-19 pandemic in Nigeria, with respect to individual- and community-level characteristics, using Bayesian hierarchical models. We used the 2018 Demographic and Health Survey as the pre-COVID-19 period and the 2021 Multiple Indicator Cluster Survey as the post-COVID-19 period.

**Results:**

At the state level, FGM prevalence varied geographically and increased by 23% and 27% in the northwestern states of Katsina and Kana, respectively. There were 11% increase in Kwara and 14% increase in Oyo. However, at the national level, the prevalence of FGM was found to decrease from 19.5% to 12.3% between 2018 and 2021. Cultural factors were identified as the key drivers of FGM among 0–14 years old girls in Nigeria. The changes in the likelihood of girls undergoing FGM across the two time periods also varied across ethnic and religious groups following COVID-19 pandemic.

**Conclusion:**

Our findings highlight that FGM is still a social norm in some states/regions and groups in Nigeria, thereby highlighting the need for a continued but accelerated FGM interventions throughout the country.

## Plain language summary

The impact of COVID-19 on the genital mutilation of girls in Nigeria

Female genital mutilation affects more than 3 million girls aged 0–14 each year. To end the practice, policymakers and non-profit organisations continue to implement community-based interventions. These interventions were interrupted during the COVID-19 pandemic as resources were diverted to care for those affected. In addition, the lockdown and closure of schools reduced the number of safe places available to girls, increasing violence against children and their vulnerability to perpetrators. Our aim was to assess the impact of COVID-19 on progress towards zero FGM. By building several statistical models, we found that while national prevalence declined over the COVID-19 period (from 19% to 12%), there were large disparities between states (+27% in Katsina in the north) and ethnic and religious groups. We also found that individual drivers of FGM for girls are more important than community drivers. Our findings support the continuation of FGM interventions across the country.

## Introduction

Female genital mutilation (FGM) is a practice involving the partial or complete removal of the external female genitalia for no medical reason.^1–3^ This practice is known to have adverse effects on both the psychological and physical health of women as FGM practices are painful and traumatic, including an increase in neonatal deaths compared to women who have not undergone any form of FGM.^1,3,4^ The practice of FGM has its roots in ancient community practices and is seen as a way to control sexual behaviour of women and ensure purity before marriage.^5^ While the practice has been reduced in several countries around the world thanks to the efforts of local communities, governments, national and international organisations,^6^ UNICEF estimates that about 200 million girls and women have undergone at least one form of FGM, with large disparities across world regions and religious, social and cultural groups.^2,3,7^ FGM practice is indeed highly influenced by local and cultural practices, resulting in social sanctions against women who are not cut, including immediate divorce, forced excision, curses and ancestral wrath.^8^ Furthermore, the community enforcement mechanism described in Mberu^8^ shows that once girls are cut, they are rewarded with public recognition and gifts, and are seen as women, allowing them to participate in adult social functions.

Recent estimates by the World Health Organisation show that 30 million girls aged 0–15 years are at risk of undergoing FGM in the next decade.^9^ Although the global prevalence of FGM is declining, this is not enough to meet the United Nations Sustainable Development Goal (SDG) 5.3 on gender equality, which calls for an end to the practice and other forms of gender-based violence by 2030.^7,10^ In addition, the global downward trend in the practice of FGM may have been interrupted in recent years by the COVID-19 crisis in 2020, which resulted in lockdowns, school closures and the diversion of health resources.^11^ Research has shown that in South Africa, lockdowns and the associated economic consequences (i.e. loss of work and reduced income) have led to food insecurity and physical violence against children, particularly girls.^12,13^ Former cutters who had abandoned the practice, as well as new cutters, turned to the practice in hopes of coping with the loss of income.^14^ This loss of income in households with children may also have led to early marriage of girls to earn money, thereby increasing FGM of girls as a prerequisite for marriage.^11^ Surveys conducted in East and West Africa have shown that the lockdown was seen by practitioners as an undetected way of performing FGM on girls.^11^ At the onset of the COVID-19 pandemic, it was estimated that the pandemic would result in 2 million more cases of FGM than in a non-pandemic scenario.^15^ This potential increase in the global incidence of FGM cases must be added to the natural population increase in regions where FGM is practiced, meaning that while prevalence is decreasing, the absolute number of girls cut is increasing.^1^ In some countries, such as Nigeria, prevalence has decreased among women aged 15–49, but not among girls, with an increase from 16.9% in 2013 to 19.2% in 2018, despite a 2015 law (i.e. the Violence against Persons (Prohibition) Act) banning the practice of FGM.^16^ Furthermore, Nigeria has a high regional variation in FGM prevalence^17,18^ and a high economic burden due to the practice of FGM.^19^ This has led several authors^18,20,21^ to investigate the spatio-temporal evolution of FGM prevalence patterns using Bayesian hierarchical models.

While some qualitative studies have explored the impact of the COVID-19 pandemic on FGM prevalence in Nigeria,^11,22^ no research to date has used a Bayesian hierarchical modelling framework to examine this impact on Nigerian girls aged 0–14 years, accounting for individual- and community-level drivers of FGM and providing uncertainty around estimates of likelihood. This study aims to fill this gap by analysing how FGM prevalence and likelihood have evolved over the pandemic, using nationally representative datasets from the Demographic and Health Survey (DHS) and the Multiple Indicator Cluster Survey (MICS). We examined temporal trends in the prevalence of FGM among girls between 2018 and 2021 across different cultural, social, and geographic groups in Nigeria, controlling for both community (e.g. community support for FGM, geopolitical zone, community prevalence of FGM) and individual-level (e.g. mother’s education level, mother’s FGM status) drivers of FGM.

## Methods

### Study data and variables

We used data from the DHS conducted in Nigeria in 2018 for the pre-COVID-19 period and data from the Nigerian MICS in 2021 for the post-COVID-19 period. The MICS and the DHS used a similar sampling design strategy: primary sampling units, or clusters, were first selected with probability based on their population size, and then, a group of 25–30 households in each cluster was randomly selected. The full methodology for sampling the DHS and the MICS datasets using stratified multistage sampling, including the methodology for estimating the optimal sample size through a specific power analysis called power allocation (see section 1.6 of ICF International^23^ for DHS and UNICEF^24^ for MICS), determining sampling weights and reducing sampling error, are detailed in ICF International^23^ and in UNICEF^24^ for DHS and MICS, respectively. Women aged 15–49 in each of the selected households were interviewed using the Women’s Questionnaire (available in National Population Commission^25^ for DHS and in National Bureau of Statistics^26^ for MICS), which includes a module on FGM. Women who have ever heard of FGM were asked about their FGM status, their opinion on the continuation of the practice and the FGM status of their girls, if any, among other FGM-related questions. After data checks and cleaning, we retained data on 41,821 women and 24,143 girls aged 0–14 years from the 2018 DHS dataset collected from 1400 clusters.^25^ From the 2021 MICS, we extracted data on 40,326 women and 19,034 girls from 1755 clusters.^26^ We then followed the appropriate DHS and MICS procedures^25,26^ to obtain representative estimates of FGM-related characteristics, following other studies.^18,20,21,27^ We have followed the STROBE Guidelines developed in von Elm et al.^28^ when preparing the manuscript.

### Statistical analysis

The statistical model used in this study follows the Bayesian hierarchical regression modelling framework.^29–31^ The dependent variable was a binary variable indicating the FGM status of a Nigerian girl aged 0–14 years, taking the value of 1 if the girl had been cut and 0 if she had not been cut at the time of the survey for each dataset. Explanatory variables included individual-level characteristics of the girls and their mothers (see Table 1). We also included the sampling weights of each survey as a covariate to ensure the representativeness of the sample.

**Table 1.** Individual- and community-level characteristics investigated in this study.

Level	Characteristics
Individual	Mother education, mother age, girl age, household wealth quintile, mother marital status, ethnicity, religion, mother support for FGM continuation, mother FGM status
Community	Geopolitical zone, residence, percentage of women with at least secondary education, percentage of women supporting FGM continuation, percentage of women that are cut, EFI, main religion in community, main wealth quintile in community

FGM: female genital mutilation; EFI: Ethnic Fractionalisation Index.

**Figure 1.**
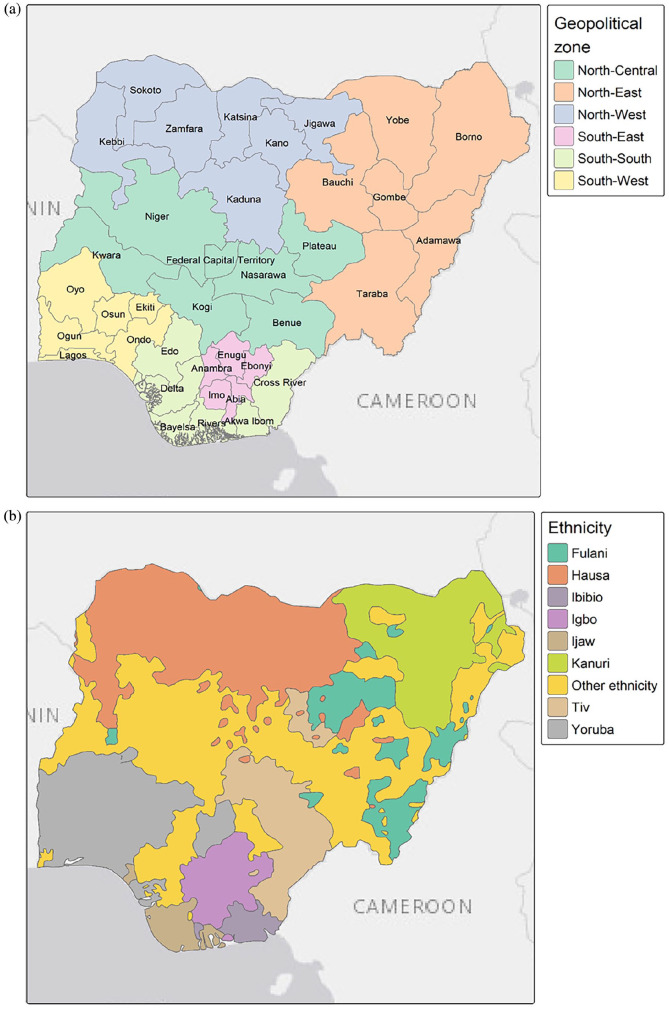
Maps of Nigeria’s states, the FCT, geopolitical zones (a) and ethnic groups (b). The administrative boundaries shapefile was downloaded from GADM. Data on ethnicity was obtained from the GREG dataset.^35^ FCT: Federal Capital Territory; GREG: geo-referencing of ethnic groups.

As the practice of FGM is also known as a social norm, in which the decisions of individuals (mothers) are influenced by the shared beliefs and practices of the majority in the community (defined here as the survey cluster),^32–34^ we also examined variables that are indicative at the community level. These included the geographic location of women and girls (i.e. geopolitical zones as shown in Figure 1(a) and urban/rural residence), as people who live closer together are more likely to be part of the same community and therefore follow similar socio-cultural norms.^20^ Other community-level variables are listed in Table 1. We also included an Ethnic Fractionalisation Index (EFI),^20^ which ranges from 0 to 1 and indicates the ethnic mix in a community, with mono-ethnic communities having an EFI close to 0 and multi-ethnic communities with groups of equal size having an EFI close to 1.

In multi-ethnic communities, it may be easier for members of the community to choose not to practise FGM if at least one of the ethnic groups has abandoned the practice. Conversely, in mono-ethnic communities where FGM is still a social norm, it may be more difficult for individuals to oppose the practice.^20^ The EFI is calculated using:

(1) $$\mathrm{EFI}=1-\sum_{k=1}^{n}s_{k}^{2}$$

where $s_{k}$ is the proportion of the *k*^th^ ethnic group in a community with n≥2 ethnic groups. Main Nigerian ethnic groups are shown in Figure 1(b). Note that the MICS religion and ethnicity variables are only collected at household level and are therefore based on the household head, whereas in the DHS, we used the mother’s religion and ethnicity.

### Bayesian hierarchical modelling

In contrast to previous work^18,20^ that used Markov chain Monte Carlo (MCMC) algorithms to model the likelihood of FGM among girls and women, we used logistic Bayesian hierarchical modelling within the integrated nested Laplace approximation (INLA),^29^ which offers an improvement over MCMC in terms of computational requirements. This allowed us to account for the effect of individual- and community-level variables and spatial autocorrelation on a girl’s FGM status, coded 0 if not cut and 1 if cut, and to generate posterior estimates of FGM prevalence including uncertainty estimates. Our Bayesian logistic model is expressed as follows:

(2) $$\begin{array}{l} \mathrm{logit}\left(p_{i}\right)=\beta_{0}+{z^{\prime }}_{i}\;\beta +f_{1}\left(x_{i1}\right)+\ldots +f_{p}\left(x_{ip}\right)\\ +f_{\mathrm{str}}\left(s_{i}\right)+f_{\mathrm{unstr}}\left(s_{i}\right)+\beta_{w}\mathrm{weight} \end{array}$$

where $p_{i}$ is the probability of girl i being cut (random variable y ) following a Bernoulli distribution, $\beta_{0}$ is the intercept, β is the vector of regression coefficients of the $z_{i}^{\prime }$ vector of covariates and $f_{1},.....,f_{p}$ are smooth functions included to account for the non-linear effect of covariates such as the girl’s current age, the age of her mother, the proportion of women cut in the community and the proportion of women in the community who support the continuation of FGM.^18,20^ To adjust for the representativeness of the sample, the sample weight (with regression coefficient $\beta_{w})$ of the survey respondents was added as a covariate. To assess the extent to which individual- and community-level characteristics influence a girl’s FGM status, we fitted several Bayesian models based on different combinations of individual- and community-level variables (see Table 1).

In addition to using individual- and community-level variables, we performed Bayesian modelling with structured $f_{\mathrm{str}}\left(s_{i}\right)$ and unstructured $f_{\mathrm{unstr}}\left(s_{i}\right)$ spatial random variation across the 37 states of residence of girls and their mothers. The structured effects account for spatial autocorrelation between neighbouring states, while the unstructured effects represent the remaining spatial variation not included in the covariates and the spatially correlated effects. The structured spatial effects of the models are expressed as an intrinsic conditional autoregressive *Besag* model.^36^ The unstructured spatial effects follow a zero-mean independent and identically distributed Gaussian prior. All model structures tested in this study are shown in Table 2.

**Table 2.** Bayesian model specifications.

Complexity	Model	Specification
Low	m1	$\beta_{0}+{z^{\prime }}_{i}\beta +f_{1}\left(x_{i1}\right)+\ldots +f_{p}\left(x_{ip}\right)+\beta_{w}\mathrm{weight}$
	m2	$\beta_{0}+{z^{\prime }}_{i}\beta +f_{1}\left(x_{i1}\right)+\ldots +f_{p}\left(x_{ip}\right)+f_{\mathrm{str}}\left(s_{i}\right)+\beta_{w}\mathrm{weight}$
High	m3	$\beta_{0}+{z^{\prime }}_{i}\beta +f_{1}\left(x_{i1}\right)+\ldots +f_{p}\left(x_{ip}\right)+f_{\mathrm{str}}\left(s_{i}\right)+f_{\mathrm{unstr}}\left(s_{i}\right)+\beta_{w}\mathrm{weight}$

To compare all models, we calculated the deviance information criteria (DIC) for m1, m2 and m3 to select the best-fitting model (i.e. the model that lowered the DIC). To compare the use of individual-, community- and both individual- and community-level variables in the models, we estimated the *R*² (i.e. the square of the correlation between observations and predictions), root-mean-square error (RMSE) and mean absolute error (MAE) between posterior estimates and weighted observed estimates of FGM prevalence aggregated at the state level. In addition, we tested the performance of the best (lowest DIC) Bayesian model using a five-fold five-repeated cross-validation and calculated accuracy (proportion of correct predictions to all predictions), precision (proportion of true positives to all predictions), recall (proportion of true positives to all positives) and the area under curve (AUC). The model estimates of the regression coefficients β are presented as posterior odd ratios (POR).

## Results

### Descriptive analysis

Over the COVID-19 pandemic period, the national prevalence of FGM among girls aged 0–14 years decreased from 19.2% to 14.1%, as calculated from DHS 2018 and MICS 2021 data, respectively. However, this national declining pattern masks heterogeneities when FGM prevalence is aggregated across individual and community-level characteristics that influence girls’ risk of undergoing FGM.

#### Individual-level characteristics

**Figure 2.**
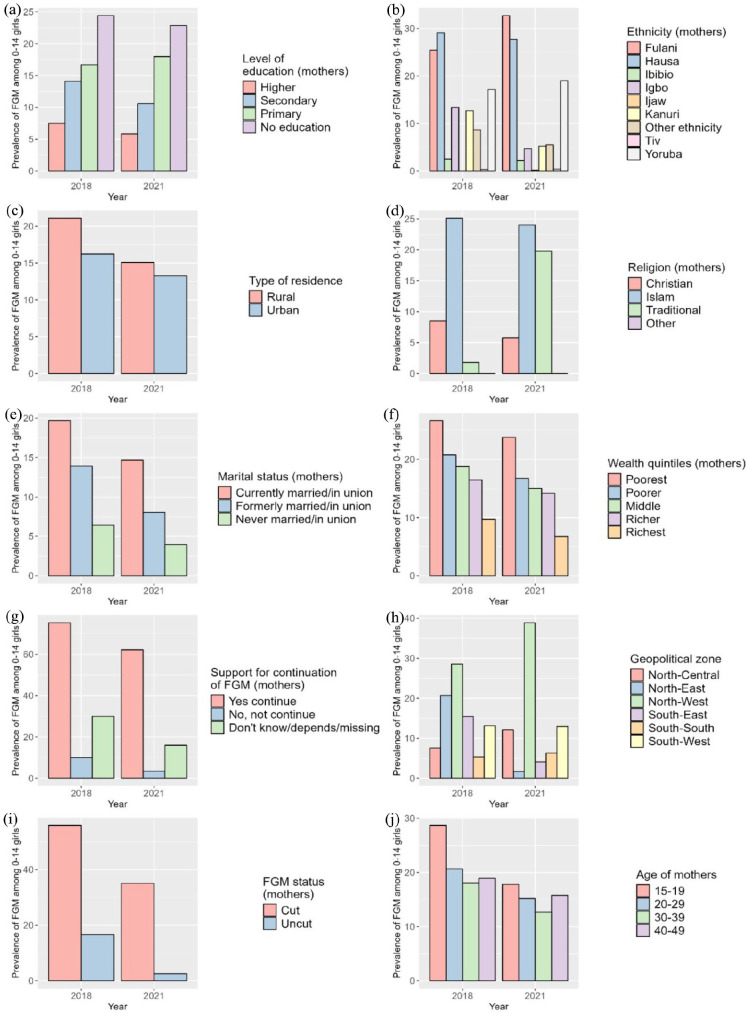
Observed FGM prevalence by individual (a, b, d, e, f, g, i, j) and community-level (c, h) characteristics in 2018 (DHS) and 2021 (MICS). FGM: female genital mutilation; DHS: Demographic and Health Survey; MICS: Multiple Indicator Cluster Survey.

Between 2018 and 2021, the prevalence of FGM among girls aged 0–14 years decreased at all levels of several individual-level variables, consistent with the overall national decline. For example, FGM prevalence decreased regardless of mothers’ marital status, but remained higher among girls whose mothers were currently in a union (i.e. 19.7% and 14.7%) compared to those formerly in a union (i.e. 13.9% and 8.1%) and never in a union (i.e. 6.5% and 3.4%) (see Figure 2 and Supplemental Table S1). FGM prevalence among girls also decreased across all age groups of their mothers, with the largest decrease among girls whose mothers were aged 15–19 years (i.e. from 28.7% to 17.8%), although this group still had the highest prevalence in 2021. In addition, girls whose mothers had undergone FGM or supported its continuation had a much higher FGM prevalence than girls whose mothers had not been cut or were against FGM, though the prevalence declined over 2018–2021. Finally, FGM prevalence decreased across all wealth quintiles, with wealthier households having lower rates in both 2018 and 2021.

While FGM prevalence decreased among daughters of mothers with no, secondary, or higher education, it increased among daughters of mothers with primary education. FGM prevalence increased among traditionalists from 18%, while it decreased among Christians and Muslims, with the latter remaining the highest practising group in 2021 (24.0%). FGM prevalence decreased among ethnic groups such as the Ibibio, Igbo and Kanuri between 2018 and 2021, but remained high among the Hausa (i.e. 29.1%–27.7%), and increased significantly among the Fulani (i.e. 25.4%–32.7%), who became the highest-practising group in 2021 (Figure 2 and Supplemental Table S1).

**Figure 3.**
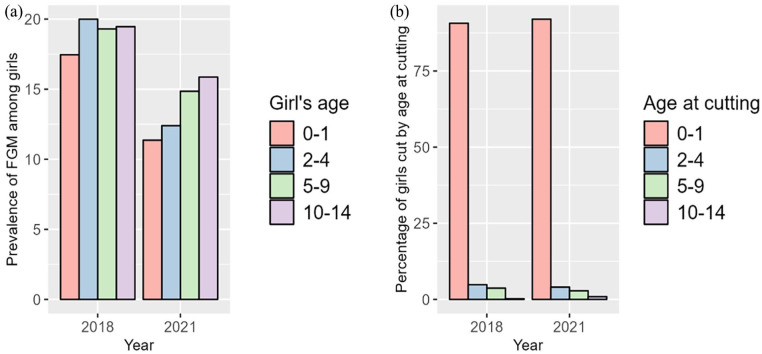
FGM prevalence by age group of girls (a) and percentage of girls cut by age at cutting (b). FGM: female genital mutilation.

FGM prevalence also varied by girls’ age, with the highest prevalence in 2018 among girls aged 2–4 years (20.0%), but shifting to girls aged 10–14 years (15.9%) by 2021 (Figure 3(a)). Despite a decrease in FGM prevalence across all age groups, the largest decrease was seen in the 2–4 year age group. Most girls continued to be cut by the age of 1 year or younger, although there was a slight increase in girls cut at 10–14 years in 2021 (Figure 3(b)).

#### Community-level characteristics

**Figure 4.**
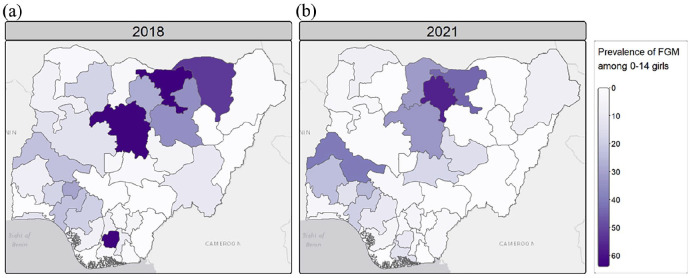
Observed prevalence of FGM in girls aged 0–14 by Nigerian states and FCT in (a) 2018 (DHS) and (b) 2021 (MICS). Shapefile of administrative units was downloaded from GADM. FGM: female genital mutilation; DHS: Demographic and Health Survey; MICS: Multiple Indicator Cluster Survey; FCT: Federal Capital Territory.

At the community level, FGM prevalence among girls aged 0–14 years showed different trends, not always in line with the national decline. For example, FGM prevalence increased among girls in the North-West (i.e. 28.6%–38.9%) and North-Central (i.e. 7.6%–12.1%) zones, while it decreased from almost 20% in the North-East zone (i.e. 20.7%–1.7%) (Figure 2 and Supplemental Table S1). In the southern areas, FGM prevalence decreased in the South-West and South-East zones but increased in the South-South zone (i.e. from 5.3% to 6.3%) between 2018 and 2021. Disparities emerged at the state level, as shown in Figure 4.

In the North-West, Jigawa and Kaduna experienced large declines (63.8%–43.6% and 63.1%–32.8%, respectively), while prevalence rose from nearly 30% in the neighbouring states of Katsina and Kano, which became the state with the highest FGM prevalence in 2021 (see Figure 4 and Supplemental Table S2). In the North-East, FGM prevalence decreased in most states, with decreases of almost 35% in Bauchi and 52% in Yobe, bringing the prevalence of FGM down to 0.0% in 2021. Different patterns are observed in the North-Central, with a decline in the practice of FGM in some states (e.g. Niger, FCT) but an increase of more than 15% in others (e.g. Kwara and Nasarawa) (see Figure 4 and Supplemental Table S2). In the South-West, states like Ondo, Osun and Lagos experienced modest declines of 4%–5%, while Oyo experienced increases of up to 15%. Most of the south-eastern states show a decrease in FGM prevalence among girls, with a sharp decrease in Imo from 62.8% to 9.8% over 2018–2021. In the South-South, FGM prevalence decreased in Edo or Akwa Ibom, but increased for girls in Rivers and Cross River (Figure 4 and Supplemental Table S2). Finally, FGM prevalence decreased in girls living in both rural and urban areas over 2018–2021, while remaining higher in rural areas (Figure 2 and Supplemental Table S1).

### Bayesian hierarchical modelling

#### Model metrics

The selection of the best fitting Bayesian model (among m1, m2 and m3) to model the likelihood of FGM was done by minimising the DIC (Table 3). Considering that a difference below 2 does not significantly affect the model fit and therefore the simpler model is better,^37^ m2 (i.e. using Besag structured spatial effect) performs better than any of the others (four times out of six models), with the use of individual-level variables providing the best fit. We calculated the $R^{2}$ , RMSE and MAE by comparing the observed and posterior predicted prevalence of FGM among girls at the state level (Table 4). The best performance is achieved with individual-level variables, with an $R^{2}$ of 0.97, indicating almost perfect agreement between observations and predictions (Supplemental Figure S1).

**Table 3.** Model DIC.

Model	DHS 2018			MICS 2021		
	m1	m2	m3	m1	m2	m3
Individual	10293.1	8710.5	8707.6	6809.7	5554.2	5555.7
Community	10670.6	10479.1	10480.1	7506.0	7119.0	7119.3
Individual and community	10862.3	10479.7	10479.9	7491.0	7015.3	7010.4

Models that best fit the data are underlined, that is, models with the lowest DIC, taking as a rule of thumb that for a difference in DIC of 2 simpler models are preferred. DIC: deviance information criteria; DHS: Demographic and Health Survey; MICS: Multiple Indicator Cluster Survey.

**Table 4.** $R^{2}$ , RMSE and MAE scores.

Model	DHS 2018			MICS 2021		
	$R^{2}$	RMSE	MAE	$R^{2}$	RMSE	MAE
Individual (m2)	0.97	3.46	2.01	0.97	2.69	1.34
Community (m2)	0.88	8.95	5.49	0.97	2.70	1.34
Individual and community (m2)	0.87	9.09	5.69	0.97	2.69	1.35

Best performing models are underlined, that is, models with the lowest RMSE, MAE and highest $R^{2}$ . RMSE, MAE and $R^{2}$ values are calculated by comparing the observed and posterior predicted FGM prevalence per state from the best fit model as indicated by the DIC in Table 3 (m2, using spatial random effects). DIC: deviance information criteria; FGM: female genital mutilation; DHS: Demographic and Health Survey; MICS: Multiple Indicator Cluster Survey; RMSE: root-mean-square error; MAE: mean absolute error.

#### FGM prevalence estimates

**Figure 5.**
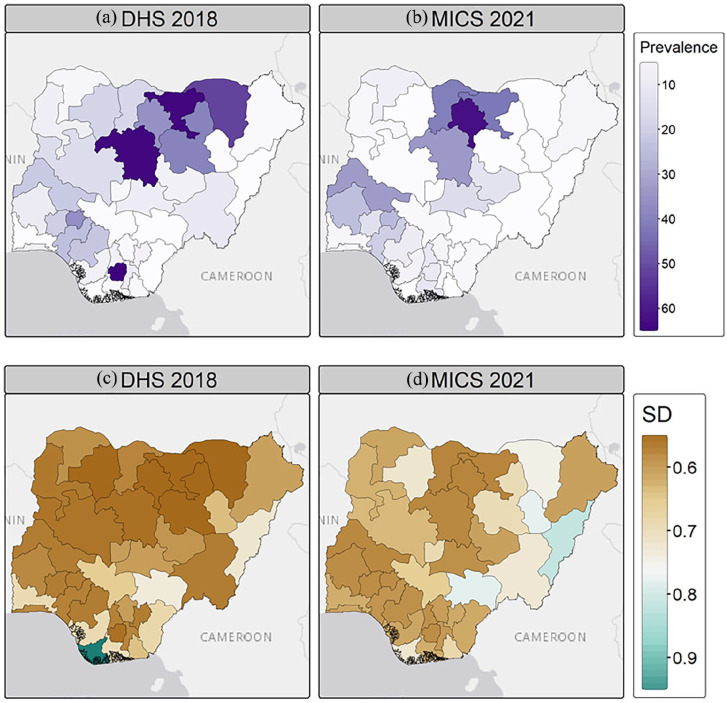
Posterior predicted FGM prevalence among girls aged 0–14 years (a, b) and uncertainty (c, d) estimates. Posterior estimates are based on the m2 model using individual-level variables and structured spatial random effects for both DHS 2018 and MICS 2021. Shapefile downloaded from GADM. SD: standard deviation; FGM: female genital mutilation; DHS: Demographic and Health Survey; MICS: Multiple Indicator Cluster Survey.

Using model m2 with structured spatial random effects and individual-level variables, we mapped the posterior estimates of FGM prevalence among girls and the standard deviations of these estimates (Figure 5). At the national level, the predicted prevalence of FGM decreased from 19.5% in 2018 to 12.3% in 2021. The standard deviation of the estimates is quite low, but slightly higher in Bayelsa in 2018 and Benue in 2021. The highest predicted prevalence is in Imo with 65.4% in 2018 and Kano with 62.3% in 2021.

**Figure 6.**
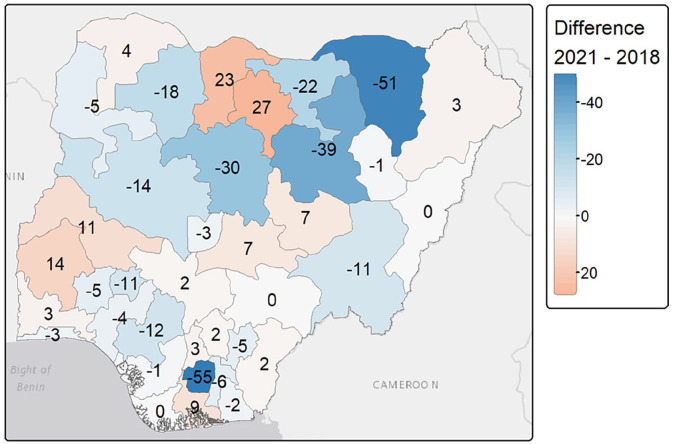
Difference in the posterior predicted FGM prevalence per state between 2021 (MICS) and 2018 (DHS). Reddish areas indicate that the FGM prevalence was higher in 2021 than in 2018, while blue areas indicate that the FGM prevalence has decreased over the period. Posterior estimates are based on the m2 model using individual-level variables and structured spatial random effects for both DHS 2018 and MICS 2021. Note that the numbers superimposed on the states are rounded to the nearest integer. FGM: female genital mutilation; DHS: Demographic and Health Survey; MICS: Multiple Indicator Cluster Survey.

At the state level, FGM prevalence shows significant heterogeneity. Some states, such as Imo (South-East) and Yobe (North-East), have seen reductions of more than 50% (see Figure 6). Neighbouring states to Imo experienced little to no change, and some, such as Rivers, actually experienced increases. In the North, Kaduna and Bauchi saw a decrease in prevalence of more than 30%, but neighbouring states such as Katsina and Kano experienced increases of more than 20%. Other increases were seen in Oyo (South-West) and Kwara (North-Central), while prevalence in neighbouring states (e.g. Niger, Ekiti) decreased between 2018 and 2021. Some states showed no change between 2018 and 2021 and kept FGM prevalence close to zero (e.g. Adamawa, Gombe, Benue). Spatial random effects are discussed in the supplementary information (see Supplemental Figure S2).

#### Cross-validation

**Figure 7.**
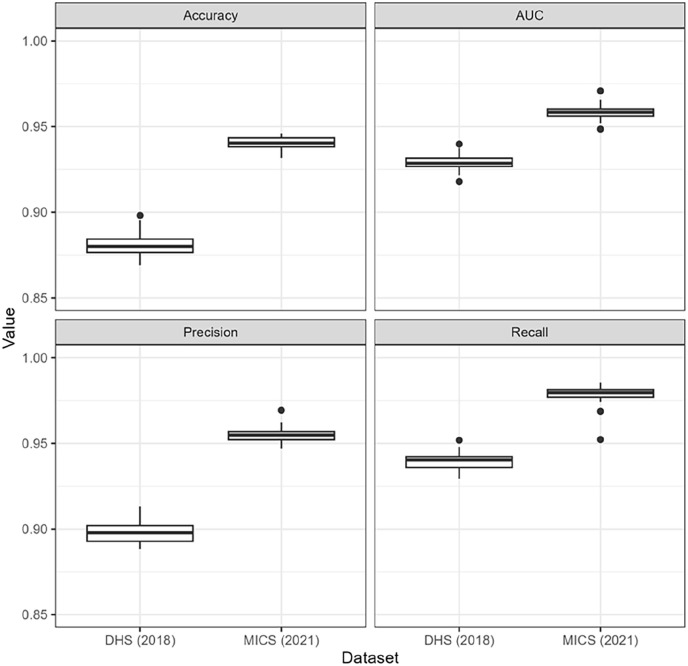
Result of the five-fold five-repeated cross-validation of model m2 using individual variables.

The performance of the best-fit model (i.e. m2) was assessed using a cross-validation framework and is presented as accuracy, AUC, precision and recall (Figure 7). Overall, all metrics show high performance, with values close to 1 and low dispersion across all 25 folds. Models using MICS data perform better than models using DHS, although they are relatively close.

#### Posterior odd ratios

We calculated the POR estimates from the best fit models, that is, m2 for both DHS 2018 and MICS 2021 (see Table 3), and presented them in Table 5.

**Table 5.** Posterior odd ratios from the Bayesian models fitted to DHS 2018 and MICS 2021 data.

Variables	Levels	DHS 2018			MICS 2021		
		POR	2.5%	97.5%	POR	2.5%	97.5%
	(Intercept)	0.022	0.014	0.034	0.0076	0.0046	0.0123
Mother education	No education (ref)	1	—	—	1	—	—
	Higher	0.708	0.522	0.961	0.8362	0.5912	1.1829
	Secondary	0.971	0.802	1.176	1.1939	0.9537	1.4947
	Primary	1.033	0.870	1.227	1.1743	0.9472	1.4557
Mother marital status	Currently married/in union (ref)	1	—	—	1	—	—
	Formerly married/in union	0.764	0.585	0.997	0.8262	0.6036	1.131
	Never married/in union	1.207	0.575	2.531	2.099	1.047	4.2078
Mother age		See Figure 8(a)			See Figure 8(b)		
Girl age		See Figure 8(c)			See Figure 8(d)		
Mother support for FGM continuation	No, not continue (ref)	1	—	—	1	—	—
	Yes continue	16.436	14.324	18.861	26.728	22.280	32.072
	Don’t know/depends/missing	2.311	1.942	2.750	3.620	2.972	4.409
Mother FGM status	Uncut (ref)	1	—	—	1	—	—
	Cut	8.145	7.022	9.461	10.992	8.991	13.439
Wealth quintile	Poorest (ref)	1	—	—	1	—	—
	Poorer	0.937	0.799	1.100	1.006	0.797	1.271
	Middle	0.952	0.797	1.137	1.060	0.831	1.354
	Richer	0.804	0.655	0.987	1.212	0.934	1.572
	Richest	0.606	0.473	0.777	0.800	0.578	1.105
Ethnicity	Fulani (ref)	1	—	—	1	—	—
	Hausa	0.930	0.768	1.127	0.478	0.356	0.643
	Ibibio	0.851	0.274	2.643	0.246	0.086	0.706
	Igbo	1.043	0.637	1.708	0.241	0.130	0.447
	Ijaw	0.000	0.000	0.000	0.016	0.003	0.078
	Kanuri	0.614	0.405	0.932	0.242	0.112	0.524
	Other	0.552	0.438	0.696	0.147	0.096	0.224
	Tiv	0.086	0.010	0.778	0.140	0.027	0.733
	Yoruba	0.809	0.541	1.210	0.654	0.419	1.021
Religion	Christian (ref)	1	—	—	1	—	—
	Islam	1.344	1.059	1.705	1.998	1.564	2.553
	Traditional	0.163	0.035	0.754	2.779	1.533	5.039
	Other	0.000	0.000	0.017	0.000	0.000	0.002
Sampling weight		1.018	0.933	1.110	0.8901	0.8209	0.9652

POR are derived from the m2 model using individual-level variables and i.i.d. spatial random effects for both DHS 2018 and MICS 2021. Underlining indicates significant relationships, that is, where the 2.5% and 97.5% CIs are both either greater or less than 1. CI: confidence interval; POR: posterior odd ratio; FGM: female genital mutilation; DHS: Demographic and Health Survey; MICS: Multiple Indicator Cluster Survey; i.i.d.: independent and identically distributed.

**Figure 8.**
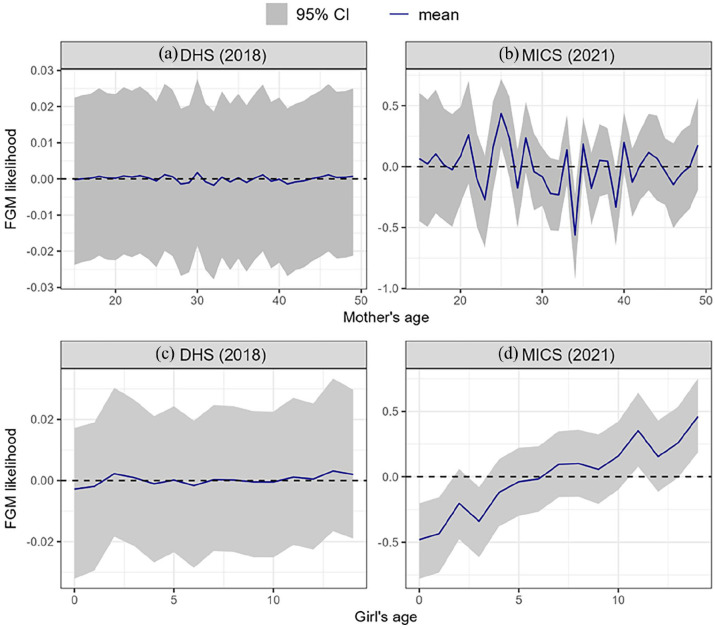
Non-linear effects (log-odds) of mother’s age (a, b) and girl’s age (c, d) on FGM likelihood. Estimates are based on the m2 model using individual-level variables and structured spatial random effects for both DHS 2018 and MICS 2021. FGM: female genital mutilation; DHS: Demographic and Health Survey; MICS: Multiple Indicator Cluster Survey.

In 2018, daughters of mothers with higher education are less likely to undergo FGM than daughters of mothers with no education (Table 5). Also in 2018, girls whose mothers were previously in a union are less likely to undergo FGM than girls whose mothers are currently married. By 2021, this relationship is no longer significant, and girls whose mothers have never been in a union are more than twice as likely to perform FGM as girls whose mothers are currently married. Mothers’ support for the continuation of FGM also strongly influence the likelihood of FGM among their daughters; girls are 16 and 27 times more likely to be cut if their mothers support the continuation of FGM in 2018 and 2021, respectively. Girls whose mothers were cut are 8 and 11 times more likely to be cut in 2018 and 2021, respectively (Table 5). In 2021, the likelihood of FGM varies strongly with mother’s age, particularly between the ages of 20 and 45, before increasing for older mothers (Figure 8(a)). FGM likelihood also rises sharply as a function of girl’s age (Figure 8(d)).

Cultural factors also influence the likelihood of FGM, with Muslim girls more likely to experience FGM than Christians in 2018 and even more so in 2021 (Table 5). Traditionalists are less likely to perform FGM in 2018, but the relationship is reversed in 2021, with girls in traditional households almost three times more likely to be cut than those in Christian households. Being from an ethnic group other than Fulani reduces the likelihood of FGM among girls, with this effect being significant for Ijaw, Kanuri, Tiv and others in 2018, and for all ethnic groups except Yoruba by 2021. Finally, girls from wealthier households are less likely to be cut in 2018, but household wealth does not affect the likelihood of FGM differently in 2021 (Table 5).

## Discussion

The lockdown during the COVID-19 pandemic led to the closure of schools and other safe places, leading to an increase in domestic violence, including against children. In addition, in Nigeria and other low-income countries, COVID-19 led to a large loss of income, resulting in food insecurity.^38,39^ Economically affected households needed other sources of income and adopted adaptation strategies to continue earning money.^38,39^ One such strategy was to marry off daughters to receive the bride price, which in turn increased FGM on girls as it is seen as a prerequisite for marriage. While qualitative research has explored these patterns through interviews with the population, there is a lack of nationally representative data and statistical analyses to quantify the impact of the COVID-19 pandemic on FGM practice. The aim of this study was to fill this gap by examining the likelihood and prevalence of FGM among girls aged 0–14 years in Nigeria, before and after COVID-19, at the national and sub-national levels. We modelled girls’ FGM status with respect to individual- and community-level FGM drivers using a Bayesian hierarchical model implemented within INLA. We also compared the added value of explicitly including unstructured and structured spatial random effects with a baseline model that included only covariates, and cross-validated the best-fit model.

Using the best-fit model, the predicted national FGM prevalence was 19.5% in 2018 and 12.3% in 2021. However, this decrease between 2021 and 2018 was not shared across religious and ethnic groups, with an increase among traditionalists and among the Fulani, Ijaw, Tiv and Yoruba. Geographically, FGM prevalence increased in three geopolitical zones: North-West, North-Central and South-South. Within these geopolitical zones, most of the increase was located in Kwara, Oyo, Katsina and Kano states. However, in some states, such as Imo (South-East) and Yobe (North-East), the prevalence of FGM decreased by more than 50%. These findings are consistent with previous work^18,27^ that highlighted strong spatial variation in FGM prevalence between 2003 and 2017 among Nigerian girls aged 0–14 years. They found that FGM prevalence, which was initially higher in southern Nigerian states, decreased in these areas between 2008 and 2017, but increased in the North-West, resulting in an average increase in prevalence at the national level (from 17.3% to 25.3% over 2003–2017). In our study, we showed that FGM is still more prevalent in the northern parts of the country, even more so after the COVID-19 period, while also highlighting some resurgence in the south.

Of all the sets of variables, we found that using only individual-level variables led to the best model fit (DIC) and validation metrics (RMSE, MAE, $R^{2}$ ) when aggregated at the states level. That is, a girl’s FGM status here was more related to the characteristics of her mother than to the characteristics of her community, whether before or after the COVID-19 pandemic. Girls whose mothers were highly educated were less likely to have undergone FGM in 2018, but not after the pandemic. In 2021, mother support for continuing FGM dramatically increased the likelihood of FGM compared to 2018, as mothers who supported FGM were 26 times more likely to have their daughters cut in 2021. This is consistent with other studies^18,21^ where support for FGM significantly increased the likelihood of FGM for girls in Nigeria, and in Ethiopia.^40^ Another highly significant factor was the FGM status of mothers, where girls were 8 times more likely to be cut in 2018 and 11 times more likely in 2021 if their mothers were also cut, supporting the social-norms theory.^21^ Similar results were found in Kenya^20^ and in Ethiopia.^40^ We also found that the likelihood of being cut was higher among never-married mothers after COVID-19 compared to currently married mothers. This could be explained by the increased vulnerability of single-income households to external changes (such as the COVID-19 pandemic), such as single mothers having to find another source of income as they do not have a husband’s salary. They may rely more on bride price as an alternative source of income and hence to marry off more of their daughters during the COVID-19 pandemic compared to currently married women.

While belonging to the traditionalist religious group reduced the likelihood of a girl being cut in 2018, the effect was reversed in 2021, when they were more than twice as likely to be cut as Christians. Similarly, surveys conducted in Abuja in 2019 found that FGM was more likely to be performed on daughters among traditionalist women than any other religious group.^41^ They also found that the FGM status of the mother affected the FGM status of a girl differently across three generations of mothers. This is consistent with our findings, as we found a strong non-linear relationship between the likelihood of a girl being cut and the age of her mother in 2021 (Figure 8(b)), with girls from younger (before 20) and older (after 40) mothers more likely to be cut. Further studies could assess the effect of a mother’s generation on the likelihood of a daughter being cut, especially as older mothers may be more open to reassessing their social norms and practices, and younger mothers may lack the moral authority to challenge beliefs around FGM,^42^ potentially leading to a tipping point in the way FGM is viewed. In our study, girls were more likely to be cut if their mother was Muslim than if she was Christian, in line with Kandala et al.^20^ Another significant variable was ethnicity, where in 2021, the likelihood of FGM was lower in all other groups than the Fulani, except for the Yoruba. In contrast, in Nnanatu et al.,^21^ the likelihood of FGM is significantly lower among the Yoruba than the Fulani. However, ethnicity is a significant variable in Nigeria for all survey years considered in Nnanatu et al.^18^

The use of unstructured spatial effects did not improve the quality of the fit compared to the use of structured spatial effects. This means that the spatial distribution of FGM prevalence is explained by spatial autocorrelation between neighbouring states rather than heterogeneity between states. This may be related to the use of some variables in the model, such as ethnicity. Indeed, ethnic groups are highly clustered in space, with some FGM-practising ethnic groups spanning several neighbouring states and using structured spatial effects can account for this spatial distribution (Figure 1). Study^43^ further highlights that the distribution of FGM in Nigeria mainly follows the distribution of ethnic groups. This may be because FGM is strongly rooted in certain ethnic groups and FGM is passed down generationally through mothers and daughters of the same ethnic group, unless there are social sanctions against women who perform FGM. This suggests that the norm in the mother’s ethnic group needs to change before there is a shift from performing FGM to not performing FGM.^44^ This is consistent with the social norm theory, which suggests that households are more likely to practice FGM if it is the norm in their community.^32–34^ Conversely, in groups where FGM prevalence is low, social pressure is expected to be lower because the reference group is one that does not cut their daughter, leading to even less FGM among daughters.^42^ Apart from ethnicity, another variable that is strongly clustered in space is access to education for children, which is between 95% and 100% in the South-South and South-East geopolitical zones, ultimately affecting school attendance and time spent in safe places (schools) for girls.^45,46^

### Limitations

This study has a few limitations. Since FGM status is recorded at the time of the survey (DHS or MICS), a girl who has not been cut may still be cut in the future. Survival analysis techniques can account for this by right-censoring girls not yet cut, providing insight into the likelihood of being cut in the future. In addition, the DHS and MICS data contain information on the FGM status of daughters as reported by their mothers. Mothers may also falsely report that their girls have undergone FGM in order to conform to community norms or to avoid repercussions.^32^ There may also be differences in reporting between urban and rural areas as a recent study in Ebonyi state showed that urban residents were more likely to report any violence, abuse and exploitation to relevant authorities during the COVID-19 period, compared to rural residents.^47^ Also, women in southern Nigeria tend to be more educated, aware of any laws against FGM and have been the focus of anti-FGM interventions, leading to potential under-reporting in these areas, whereas women in the north would be more likely to report the true rate of FGM.^21^ Finally, we note that although the DHS and MICS are very similar in terms of sampling strategy and sample composition to ensure sub-national representativeness, and have been used together in previous studies in Nigeria,^18,48^ there may still be some differences. Thus, future studies may focus on examining the differences between these two nationally representative household surveys and how they might affect the accuracy of model parameter estimates.

## Conclusion

The aim of our study was to understand the evolution of the practice of FGM among girls aged 0–14 years in Nigeria between the pre-COVID-19 period, 2018, and the post-COVID-19 period, 2021. Using a Bayesian spatial model and individual covariates, we were able to generate estimates of FGM prevalence and select significant drivers of FGM among girls in 2018 and 2021. Despite the posterior estimates showing a national decline from 19.5% in 2018 to 12.3% in 2021, several states experienced significant increases in prevalence. Thanks to two nationally representative surveys conducted across the country (DHS and MICS), we found that FGM among girls is strongly associated with individual characteristics of mothers, rather than community-level characteristics. Regarding the evolution of the drivers of FGM during the period 2018–2021, we found that while being in the two highest wealth quintiles reduced the likelihood of FGM before the COVID-19 pandemic, this was not the case after the pandemic. We also found that, compared to the Fulani, all other ethnic groups were less likely to practice FGM after COVID-19 in 2021. The study could help achieve SDG 5 on gender equality, which calls for the practice of FGM to be ended by 2030. We encourage further qualitative research on the impact of COVID-19 on FGM practice to understand the influence of confinement and loss of income on girls’ FGM status as well as the resurgence of FGM practice in several states of Nigeria.

## Supplemental Material

sj-docx-1-whe-10.1177_17455057241311948 – Supplemental material for A robust cross-sectional assessment of the impacts of COVID-19 pandemic on the prevalence of female genital mutilation among 0–14 years old girls in Nigeria Supplemental material, sj-docx-1-whe-10.1177_17455057241311948 for A robust cross-sectional assessment of the impacts of COVID-19 pandemic on the prevalence of female genital mutilation among 0–14 years old girls in Nigeria by Corentin Visée, Camille Morlighem and Chibuzor Christopher Nnanatu in Women’s Health

sj-docx-2-whe-10.1177_17455057241311948 – Supplemental material for A robust cross-sectional assessment of the impacts of COVID-19 pandemic on the prevalence of female genital mutilation among 0–14 years old girls in Nigeria Supplemental material, sj-docx-2-whe-10.1177_17455057241311948 for A robust cross-sectional assessment of the impacts of COVID-19 pandemic on the prevalence of female genital mutilation among 0–14 years old girls in Nigeria by Corentin Visée, Camille Morlighem and Chibuzor Christopher Nnanatu in Women’s Health

## Declarations

**Ethics approval and consent to participate:** Permission to use the DHS data for Nigeria (2018) was granted by the DHS Programme upon registration and request at https://dhsprogram.com/data/dataset_admin/login_main.cfm. Permission to use MICS data for Nigeria (2021) was granted by the MICS program upon registration and request at https://mics.unicef.org/visitors/sign-in.

**Consent for publication:** Not applicable.

**Availability of data and materials:** DHS 2018 and MICS 2021 data for Nigeria can be accessed through the DHS (https://dhsprogram.com/data/dataset_admin/login_main.cfm) and MICS (https://mics.unicef.org/visitors/sign-in) programmes respectively.
