# Supplementary material for: A robust cross-sectional assessment of the impacts of COVID-19 pandemic on the prevalence of female genital mutilation among 0–14 years old girls in Nigeria
Source: Womens Health (Lond). 2025 May 26;21:17455057241311948. doi: 10.1177/17455057241311948 (PMC12106985; doi:10.1177/17455057241311948)
Supplement: sj-docx-2-whe-10.1177_17455057241311948 – Supplemental material for A robust cross-sectional assessment of the impacts of COVID-19 pandemic on the prevalence of female genital mutilation among 0–14 years old girls in Nigeria [file sj-docx-2-whe-10.1177_17455057241311948.docx]

# Supplementary information

**Table S1**. FGM prevalence by individual- and community-level characteristics in 2018 (DHS) and 2021 (MICS).

| **Variable** | **Levels** | **DHS 2018 (%)** | **MICS 2021 (%)** |
| --- | --- | --- | --- |
| Geopolitical zone | North-Central | 7.6 | 12.1 |
|  | North-East | 20.7 | 1.7 |
|  | North-West | 28.6 | 38.9 |
|  | South-East | 15.5 | 4.1 |
|  | South-South | 5.3 | 6.3 |
|  | South-West | 13.2 | 12.9 |
| Mother education | No education | 24.4 | 22.8 |
|  | Primary | 16.7 | 18.0 |
|  | Secondary | 14.1 | 10.6 |
|  | Higher | 7.5 | 5.8 |
| Ethnicity | Fulani | 25.4 | 32.7 |
|  | Hausa | 29.1 | 27.7 |
|  | Ibibio | 2.5 | 2.2 |
|  | Igbo | 13.5 | 4.7 |
|  | Ijaw | 0.0 | 0.1 |
|  | Kanuri | 12.7 | 5.2 |
|  | Other ethnicity | 8.6 | 5.5 |
|  | Tiv | 0.3 | 0.4 |
|  | Yoruba | 17.2 | 19.0 |
| Mother marital status | Currently married/in union | 19.7 | 14.7 |
|  | Formerly married/in union | 13.9 | 8.1 |
|  | Never married/in union | 6.5 | 3.4 |
| Residence | Urban | 16.3 | 13.3 |
|  | Rural | 21.1 | 15.1 |
| Wealth quintile | Richest | 9.8 | 6.7 |
|  | Richer | 16.4 | 14.2 |
|  | Middle | 18.8 | 15.0 |
|  | Poorer | 20.8 | 16.7 |
|  | Poorest | 26.6 | 23.8 |
| Religion | Christian | 8.5 | 5.8 |
|  | Islam | 25.1 | 24.0 |
|  | Traditional | 1.8 | 19.8 |
|  | Other | 0.0 | 0.0 |
| Mother age | 15-19 | 28.7 | 17.8 |
|  | 20-29 | 20.7 | 15.2 |
|  | 30-39 | 18.0 | 12.7 |
|  | 40-49 | 18.9 | 15.8 |
| Mother support for FGM continuation | No, not continue | 10.0 | 3.4 |
|  | Yes continue | 75.2 | 62.3 |
|  | Don't know/depends/missing | 30.0 | 16.0 |
| Mother FGM status | Uncut | 16.6 | 2.6 |
|  | Cut | 55.9 | 35.1 |
|  | Missing | 2.91 | 30.2 |
| Girl age | 0-1 | 17.5 | 11.4 |
|  | 2-4 | 20.0 | 12.4 |
|  | 5-9 | 19.3 | 14.9 |
|  | 10-14 | 19.5 | 15.9 |

**Table S2.** Observed prevalence of FGM in Nigeria's 36 states and FCT in 2018 (DHS) and 2021 (MICS)

| **State** | **DHS 2018 (%)** | **MICS 2021 (%)** | **Difference** |
| --- | --- | --- | --- |
| Abia | 8.5 | 3.2 | -5.3 |
| Adamawa | 0.0 | 0.0 | 0.0 |
| Akwa Ibom | 1.7 | 0.5 | -1.2 |
| Anambra | 7.6 | 2.2 | -5.4 |
| Bauchi | 34.9 | 0.0 | -34.9 |
| Bayelsa | 0.0 | 0.2 | 0.2 |
| Benue | 0.2 | 0.0 | -0.2 |
| Borno | 1.1 | 7.3 | 6.2 |
| Cross River | 0.2 | 2.6 | 2.4 |
| Delta | 6.1 | 5.0 | -1.1 |
| Ebonyi | 5.2 | 0.2 | -5.0 |
| Edo | 18.7 | 9.7 | -9.0 |
| Ekiti | 30.0 | 25.1 | -4.9 |
| Enugu | 1.5 | 4.4 | 2.9 |
| Federal Capital Territory | 2.6 | 0.2 | -2.4 |
| Gombe | 0.2 | 0.0 | -0.2 |
| Imo | 62.8 | 9.8 | -53.0 |
| Jigawa | 63.8 | 43.6 | -20.2 |
| Kaduna | 63.1 | 32.8 | -30.3 |
| Kano | 27.6 | 56.3 | 28.7 |
| Katsina | 2.6 | 31.9 | 29.3 |
| Kebbi | 6.3 | 2.8 | -3.5 |
| Kogi | 0.0 | 2.4 | 2.4 |
| Kwara | 22.7 | 40.2 | 17.5 |
| Lagos | 12.7 | 4.3 | -8.4 |
| Nasarawa | 2.0 | 17.1 | 15.1 |
| Niger | 12.9 | 0.8 | -12.1 |
| Ogun | 0.0 | 3.4 | 3.4 |
| Ondo | 22.8 | 18.9 | -3.9 |
| Osun | 17.5 | 12.9 | -4.6 |
| Oyo | 8.2 | 23.1 | 14.9 |
| Plateau | 4.8 | 14.5 | 9.7 |
| Rivers | 2.1 | 12.6 | 10.5 |
| Sokoto | 2.4 | 7.5 | 5.1 |
| Taraba | 10.5 | 0.3 | -10.2 |
| Yobe | 51.8 | 0.0 | -51.8 |
| Zamfara | 17.5 | 0.0 | -17.5 |

*Note. “*Difference” column indicates the difference between the FGM prevalence in 2021 and 2018.


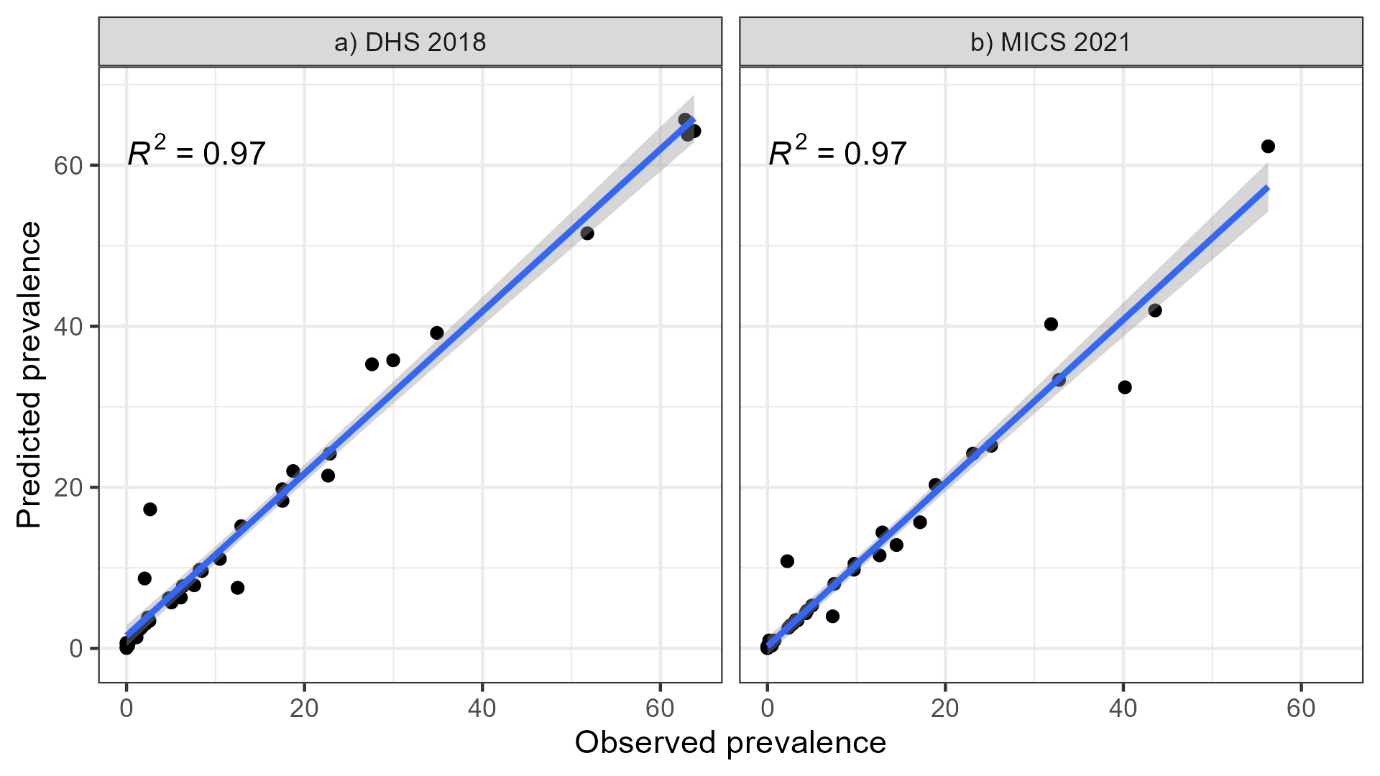


Figure S1: Observed and predicted regional prevalence of FGM among girls aged 0-14 years

### *Spatial random effects*

The latent effect of space (at state level) on FGM prevalence can be assessed by plotting the posterior spatial effects and their 95% credible intervals (Figure S2). There are more significant positive effects of neighbouring states in the DHS data than in the MICS data (17 states for the DHS compared with 15 states for MICS). This means that we observed a higher prevalence of FGM than would be expected from the covariates, mainly due to neighbouring states. In addition, states that change their significant relationship are mostly located in the northern part of the country, indicating a change in the spatial pattern of FGM prevalence. In the southern areas, the number of significant negative spatial effects decreases compared to COVID-19, apart from Abia. It should also be noted that the highest standard deviations are found in Yobe, Gombe, Adamawa and Nasarawa states. The first three states show the strongest negative relationship in the northern states.


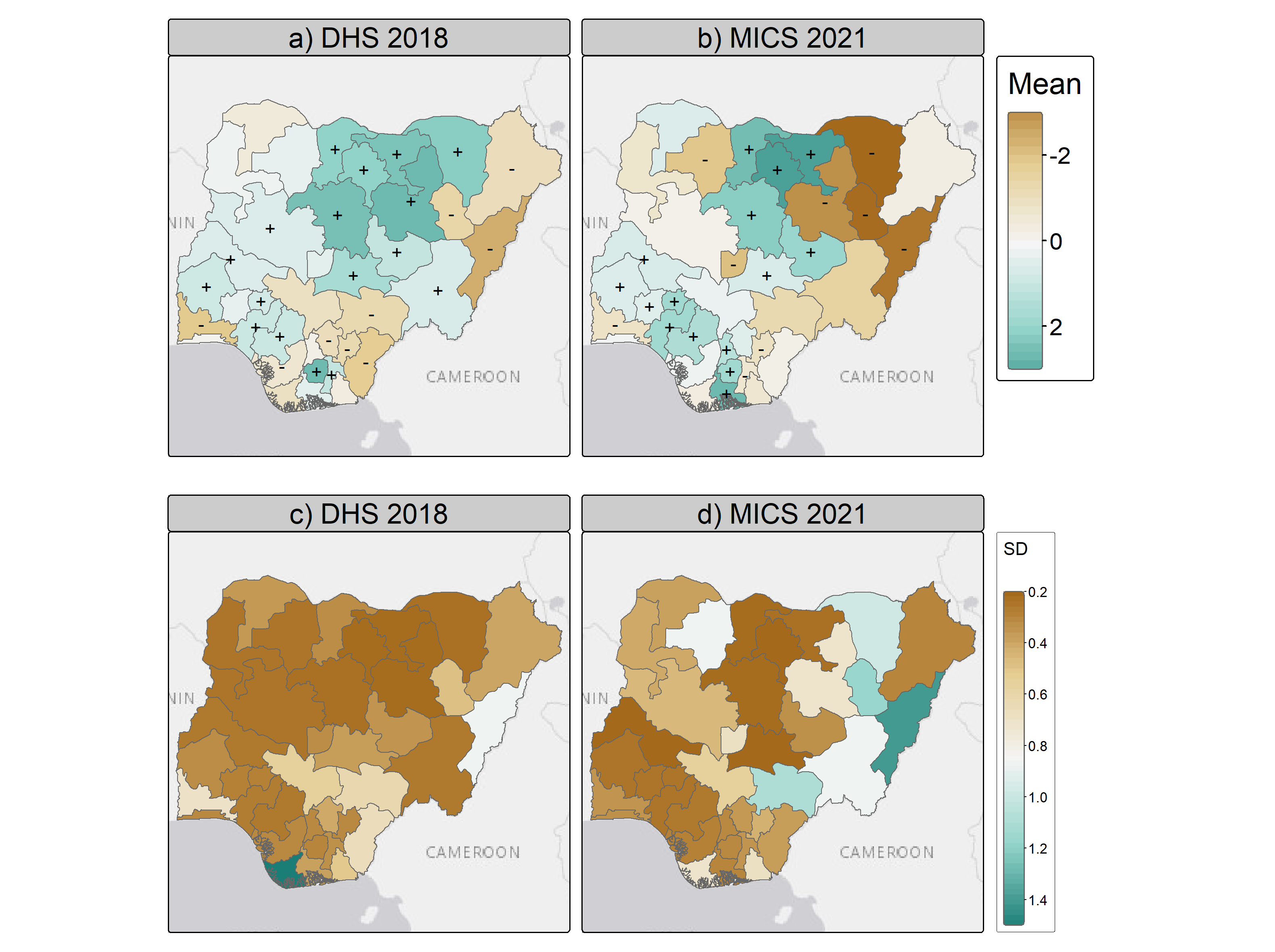


**Figure S2**: Mean spatially correlated random effect (a, b) in m2 and associated uncertainty estimates (c, d). Significant effects, based on the 95% credible interval, are shown as + if both intervals are greater than 0 and as - if the intervals are less than 0.
